# Supplementary material for: Mapping the evolution of fertility support policies in China: A content and instrumental analysis
Source: PLoS One. 2025 Oct 9;20(10):e0332137. doi: 10.1371/journal.pone.0332137 (PMC12510515; doi:10.1371/journal.pone.0332137)
Supplement: S1 Appendix — (ZIP) [file pone.0332137.s001.zip › S1 Appendix. 226 original policy documents/42-国家卫生健康委关于印发托育机构设置标准(试行)和托育机构管理规范(试行)的通知(FBM-CLI-4-336546).doc]

国家卫生健康委关于印发托育机构设置标准(试行)和托育机构管理规范(试行)的通知

**发布部门：** [国家卫生健康委员会](https://www.pkulaw.com/law/chl?Aggs.IssueDepartment=60321) [机构沿革](javascript:void(0))

**发文字号：**国卫人口发〔2019〕58号

**发布日期：**2019.10.08

**实施日期：**2019.10.08

**时效性：** [现行有效](https://www.pkulaw.com/law/chl?Aggs.TimelinessDic=01)

**效力级别：** [部门规范性文件](https://www.pkulaw.com/law/chl?Aggs.EffectivenessDic=XE0302)

**法规类别：** [卫生综合规定](https://www.pkulaw.com/law/chl?Aggs.Category=09601)

国家卫生健康委关于印发托育机构设置标准（试行）和托育机构管理规范（试行）的通知
（国卫人口发〔2019〕58号）

各省、自治区、直辖市及新疆生产建设兵团卫生健康委：
　　为加强托育机构专业化、规范化建设，按照《[国务院办公厅关于促进3岁以下婴幼儿照护服务发展的指导意见](https://www.pkulaw.com/chl/434b24b8b5769383bdfb.html)》（国办发〔2019〕15号）的要求，我委组织制定了《托育机构设置标准（试行）》和《托育机构管理规范（试行）》（可从国家卫生健康委网站下载）。现印发给你们，请遵照执行。
　　附件：1.托育机构设置标准（试行）
　　2.托育机构管理规范（试行）

国家卫生健康委
2019年10月8日

　　附件1

　　托育机构设置标准（试行）

　第一章　总则

　　第一条　为建立专业化、规范化的托育机构，根据《中华人民共和国未成年人保护法》等法律法规以及《[国务院办公厅关于促进3岁以下婴幼儿照护服务发展的指导意见](https://www.pkulaw.com/chl/434b24b8b5769383bdfb.html)》，制定本标准。

　　第二条　坚持政策引导、普惠优先、安全健康、科学规范、属地管理、分类指导的原则，充分调动社会力量积极性，大力发展托育服务。

　　第三条　本标准适用于经有关部门登记、卫生健康部门备案，为3岁以下婴幼儿提供全日托、半日托、计时托、临时托等托育服务的机构。

　第二章　设置要求

　　第四条　托育机构设置应当综合考虑城乡区域发展特点，根据经济社会发展水平、工作基础和群众需求，科学规划，合理布局。

　　第五条　新建居住区应当规划建设与常住人口规模相适应的托育机构。老城区和已建成居住区应当采取多种方式完善托育机构，满足居民需求。

　　第六条　城镇托育机构建设要充分考虑进城务工人员随迁婴幼儿的照护服务需求。

　　第七条　在农村社区综合服务设施建设中，应当统筹考虑托育机构建设。

　　第八条　支持用人单位以单独或联合其他单位共同举办的方式，在工作场所为职工提供福利性托育服务，有条件的可向附近居民开放。

　　第九条　鼓励通过市场化方式，采取公办民营、民办公助等多种形式，在就业人群密集的产业聚集区域和用人单位建设完善托育机构。

　　第十条　发挥城乡社区公共服务设施的婴幼儿照护服务功能，加强社区托育机构与社区服务中心（站）及社区卫生、文化、体育等设施的功能衔接。

　第三章　场地设施

　　第十一条　托育机构应当有自有场地或租赁期不少于3年的场地。

　　第十二条　托育机构的场地应当选择自然条件良好、交通便利、符合卫生和环保要求的建设用地，远离对婴幼儿成长有危害的建筑、设施及污染源，满足抗震、防火、疏散等要求。

　　第十三条　托育机构的建筑应当符合有关工程建设国家标准、行业标准，设置符合标准要求的生活用房，根据需要设置服务管理用房和供应用房。

　　第十四条　托育机构的房屋装修、设施设备、装饰材料等，应当符合国家相关安全质量标准和环保标准，并定期进行检查维护。

　　第十五条　托育机构应当配备符合婴幼儿月龄特点的家具、用具、玩具、图书和游戏材料等，并符合国家相关安全质量标准和环保标准。

　　第十六条　托育机构应当设有室外活动场地，配备适宜的游戏设施，且有相应的安全防护设施。
　　在保障安全的前提下，可利用附近的公共场地和设施。

　　第十七条　托育机构应当设置符合标准要求的安全防护设施设备。

　第四章　人员规模

　　第十八条　托育机构应当根据场地条件，合理确定收托婴幼儿规模，并配置综合管理、保育照护、卫生保健、安全保卫等工作人员。
　　托育机构负责人负责全面工作，应当具有大专以上学历、有从事儿童保育教育、卫生健康等相关管理工作3年以上的经历，且经托育机构负责人岗位培训合格。
　　保育人员主要负责婴幼儿日常生活照料，安排游戏活动，促进婴幼儿身心健康，养成良好行为习惯。保育人员应当具有婴幼儿照护经验或相关专业背景，受过婴幼儿保育相关培训和心理健康知识培训。
　　保健人员应当经过妇幼保健机构组织的卫生保健专业知识培训合格。
　　保安人员应当取得公安机关颁发的《保安员证》，并由获得公安机关《保安服务许可证》的保安公司派驻。

　　第十九条　托育机构一般设置乳儿班（6-12个月，10人以下）、托小班（12-24个月，15人以下）、托大班（24-36个月，20人以下）三种班型。
　　18个月以上的婴幼儿可混合编班，每个班不超过18人。
　　每个班的生活单元应当独立使用。

　　第二十条　合理配备保育人员，与婴幼儿的比例应当不低于以下标准：乳儿班1∶3，托小班1∶5，托大班1∶7。

　　第二十一条　按照有关托儿所卫生保健规定配备保健人员、炊事人员。

　　第二十二条　独立设置的托育机构应当至少有1名保安人员在岗。

　第五章　附则

　　第二十三条　各省、自治区、直辖市卫生健康行政部门可根据本标准制订具体实施办法。

　　第二十四条　本标准自发布之日起施行。
　　附件2

　　托育机构管理规范（试行）

　第一章　总则

　　第一条　为加强托育机构管理，根据《中华人民共和国未成年人保护法》等法律法规以及《[国务院办公厅关于促进3岁以下婴幼儿照护服务发展的指导意见](https://www.pkulaw.com/chl/434b24b8b5769383bdfb.html)》，制定本规范。

　　第二条　坚持儿童优先的原则，尊重婴幼儿成长特点和规律，最大限度地保护婴幼儿，确保婴幼儿的安全和健康。

　　第三条　本规范适用于经有关部门登记、卫生健康部门备案，为3岁以下婴幼儿提供全日托、半日托、计时托、临时托等托育服务的机构。

　第二章　备案管理

　　第四条　托育机构登记后，应当向机构所在地的县级以上卫生健康部门备案，提交评价为“合格”的《托幼机构卫生评价报告》、消防安全检查合格证明、场地证明、工作人员资格证明等材料，填写备案书（见附件1）和承诺书（见附件2）。提供餐饮服务的，应当提交《食品经营许可证》。

　　第五条　卫生健康部门应当对申请备案的托育机构提供备案回执（见附件3）和托育机构基本条件告知书（见附件4）。

　　第六条　托育机构变更备案事项的，应当向原备案部门办理变更备案。

　　第七条　托育机构终止服务的，应当妥善安置收托的婴幼儿和工作人员，并办理备案注销手续。

　　第八条　卫生健康部门应当将托育服务有关政策规定、托育机构备案要求、托育机构有关信息在官方网站公开，接受社会查询和监督。

　第三章　收托管理

　　第九条　婴幼儿父母或监护人（以下统称婴幼儿监护人）应当主动向托育机构提出入托申请，并提交真实的婴幼儿及其监护人的身份证明材料。

　　第十条　托育机构应当与婴幼儿监护人签订托育服务协议，明确双方的责任、权利义务、服务项目、收费标准以及争议纠纷处理办法等内容。

　　第十一条　婴幼儿进入托育机构前，应当完成适龄的预防接种，经医疗卫生机构健康检查合格后方可入托；离开机构3个月以上的，返回时应当重新进行健康检查。

　　第十二条　托育机构应当建立收托婴幼儿信息管理制度，及时采集、更新，定期向备案部门报送。

　　第十三条　托育机构应当建立与家长联系的制度，定期召开家长会议，接待来访和咨询，帮助家长了解保育照护内容和方法。
　　托育机构应当成立家长委员会，事关婴幼儿的重要事项，应当听取家长委员会的意见和建议。
　　托育机构应当建立家长开放日制度。

　　第十四条　托育机构应当加强与社区的联系与合作，面向社区宣传科学育儿知识，开展多种形式的服务活动，促进婴幼儿早期发展。

　　第十五条　托育机构应当建立信息公示制度，定期公示收费项目和标准、保育照护、膳食营养、卫生保健、安全保卫等情况，接受监督。

　第四章　保育管理

　　第十六条　托育机构应当科学合理安排婴幼儿的生活，做好饮食、饮水、喂奶、如厕、盥洗、清洁、睡眠、穿脱衣服、游戏活动等服务。

　　第十七条　托育机构应当顺应喂养，科学制定食谱，保证婴幼儿膳食平衡。有特殊喂养需求的，婴幼儿监护人应当提供书面说明。

　　第十八条　托育机构应当保证婴幼儿每日户外活动不少于2小时，寒冷、炎热季节或特殊天气情况下可酌情调整。

　　第十九条　托育机构应当以游戏为主要活动形式，促进婴幼儿在身体发育、动作、语言、认知、情感与社会性等方面的全面发展。

　　第二十条　游戏活动应当重视婴幼儿的情感变化，注重与婴幼儿面对面、一对一的交流互动，动静交替，合理搭配多种游戏类型。

　　第二十一条　托育机构应当提供适宜刺激，丰富婴幼儿的直接经验，支持婴幼儿主动探索、操作体验、互动交流和表达表现，发挥婴幼儿的自主性，保护婴幼儿的好奇心。

　　第二十二条　托育机构应当建立照护服务日常记录和反馈制度，定期与婴幼儿监护人沟通婴幼儿发展情况。

　第五章　健康管理

　　第二十三条　托育机构应当按照有关托儿所卫生保健规定，完善相关制度，切实做好婴幼儿和工作人员的健康管理，做好室内外环境卫生。

　　第二十四条　托育机构应当坚持晨午检和全日健康观察，发现婴幼儿身体、精神、行为异常时，应当及时通知婴幼儿监护人。

　　第二十五条　托育机构发现婴幼儿遭受或疑似遭受家庭暴力的，应当依法及时向公安机关报案。

　　第二十六条　婴幼儿患病期间应当在医院接受治疗或在家护理。

　　第二十七条　托育机构应当建立卫生消毒和病儿隔离制度、传染病预防和管理制度，做好疾病预防控制和婴幼儿健康管理工作。

　　第二十八条　托育机构工作人员上岗前，应当经医疗卫生机构进行健康检查，合格后方可上岗。
　　托育机构应当组织在岗工作人员每年进行1次健康检查。在岗工作人员患有传染性疾病的，应当立即离岗治疗；治愈后，须持病历和医疗卫生机构出具的健康合格证明，方可返岗工作。

　第六章　安全管理

　　第二十九条　托育机构应当落实安全管理主体责任，建立健全安全防护措施和检查制度，配备必要的安保人员和物防、技防设施。

　　第三十条　托育机构应当建立完善的婴幼儿接送制度，婴幼儿应当由婴幼儿监护人或其委托的成年人接送。

　　第三十一条　托育机构应当制订重大自然灾害、传染病、食物中毒、踩踏、火灾、暴力等突发事件的应急预案，定期对工作人员进行安全教育和突发事件应急处理能力培训。
　　托育机构应当明确专兼职消防安全管理人员及管理职责，加强消防设施维护管理，确保用火用电用气安全。
　　托育机构工作人员应当掌握急救的基本技能和防范、避险、逃生、自救的基本方法，在紧急情况下必须优先保障婴幼儿的安全。

　　第三十二条　托育机构应当建立照护服务、安全保卫等监控体系。监控报警系统确保24小时设防，婴幼儿生活和活动区域应当全覆盖。
　　监控录像资料保存期不少于90日。

　第七章　人员管理

　　第三十三条　托育机构工作人员应当具有完全民事行为能力和良好的职业道德，热爱婴幼儿，身心健康，无虐待儿童记录，无犯罪记录，并符合国家和地方相关规定要求的资格条件。

　　第三十四条　托育机构应当建立工作人员岗前培训和定期培训制度，通过集中培训、在线学习等方式，不断提高工作人员的专业能力、职业道德和心理健康水平。

　　第三十五条　托育机构应当加强工作人员法治教育，增强法治意识。对虐童等行为实行零容忍，一经发现，严格按照有关法律法规和规定，追究有关负责人和责任人的责任。

　　第三十六条　托育机构应当依法与工作人员签订劳动合同，保障工作人员的合法权益。

　第八章　监督管理

　　第三十七条　托育机构应当加强党组织建设，积极支持工会、共青团、妇联等组织开展活动。
　　托育机构应当建立工会组织或职工代表大会制度，依法加强民主管理和监督。

　　第三十八条　托育机构应当制订年度工作计划，每年年底向卫生健康部门报告工作，必要时随时报告。

　　第三十九条　各级妇幼保健、疾病预防控制、卫生监督等机构应当按照职责加强对托育机构卫生保健工作的业务指导、咨询服务和监督执法。

　　第四十条　建立托育机构信息公示制度和质量评估制度，实施动态管理，加强社会监督。

　第九章　附则

　　第四十一条　各省、自治区、直辖市卫生健康行政部门可根据本规范制订具体实施办法。

　　第四十二条　本规范自发布之日起施行。
　　附件：1.托育机构备案书
　　2.备案承诺书
　　3.托育机构备案回执
　　4.托育机构基本条件告知书
　　附件1

　　托育机构备案书

________卫生健康委（局）：
　　经________（登记机关名称）批准，________（托育机构名称）已于____年____月____日依法登记成立，现向你委（局）进行备案。本机构备案信息如下：
　　机构名称：
　　机构住所：
　　登记机关：
　　统一社会信用代码：
　　机构负责人姓名：
　　机构负责人身份证件号码：
　　机构性质：□营利性□非营利性
　　服务范围：□全日托□半日托□计时托□临时托
　　服务场所性质：□自有□租赁
　　机构建筑面积：
　　室内使用面积：
　　室外活动场地面积：
　　收托规模：人
　　编班类型：□乳儿班□托小班□托大班□混合编班
　　联系人：
　　联系方式：
　　请予以备案。

备案单位：（章）
年　月　日

　　附件2

　　备案承诺书

　　本单位承诺如实填报备案信息，并将按照有关要求，及时、准确报送后续重大事项变更信息。
　　承诺已了解托育机构管理相关法律法规和标准规范，承诺开展的服务符合《托育机构基本条件告知书》要求。
　　承诺按照诚实信用、安全健康、科学规范、儿童优先的原则和相关标准及规定，开展3岁以下婴幼儿托育服务，不以托育机构名义从事虐待伤害婴幼儿、不正当关联交易等损害婴幼儿及其监护人合法权益和公平竞争市场秩序的行为。
　　承诺主动接受并配合卫生健康部门和其他有关部门的指导、监督和管理。
　　承诺不属实，或者违反上述承诺的，依法承担相应法律责任。

备案单位：（章）
机构负责人签字：
年　月　日

　　附件3

　　托育机构备案回执

　　编号：＿＿＿＿＿＿＿＿＿＿
　　＿＿＿＿＿年＿＿＿＿＿月＿＿＿＿＿日报我委（局）的《托育机构备案书》收到并已备案。
　　备案项目如下：
　　机构名称：
　　机构住所：
　　机构性质：
　　机构负责人姓名：

＿＿＿卫生健康委（局）（章）
年　月　日

　　附件4

　　托育机构基本条件告知书

　　托育机构应当依照相关法律法规和标准规范开展服务活动，并符合下列基本条件：

　　一、应当符合《中华人民共和国未成年人保护法》《[中华人民共和国建筑法](https://www.pkulaw.com/chl/bab044f0521540e6bdfb.html)》《[中华人民共和国消防法](https://www.pkulaw.com/chl/2dd465b94d9c3a4bbdfb.html)》《[托儿所幼儿园卫生保健管理办法](https://www.pkulaw.com/chl/c2e282d5a2ded4d9bdfb.html)》等法律法规，以及《托儿所、幼儿园建筑设计规范》《[建筑设计防火规范](https://www.pkulaw.com/chl/43869fc3bf1af0c5bdfb.html)》等国家标准或者行业标准。

　　二、应当符合《托育机构设置标准（试行）》《托育机构管理规范（试行）》等要求。

　　三、提供餐饮服务的，应当符合《[中华人民共和国食品安全法](https://www.pkulaw.com/chl/15ad8fe631e24d52bdfb.html)》等法律法规，以及相应的食品安全标准。

　　四、法律法规规定的其他条件。

© 北大法宝：（[www.pkulaw.com](https://www.pkulaw.com/)) 专业提供法律信息、法学知识和法律软件领域各类解决方案。北大法宝为您提供丰富的参考资料，正式引用法规条文时请与标准文本核对。
欢迎查看所有[产品和服务](https://www.pkulaw.com/helps/2.html)。法宝快讯：[如何快速找到您需要的检索结果？](https://www.pkulaw.com/helps/70.html)[法宝](https://www.pkulaw.com/helps/69.html)V6有何新特色？

扫描二维码阅读原文

**原文链接：**<https://www.pkulaw.com/chl/bc9a2a9250f37a24bdfb.html>
